# Supplementary material for: Uncovering the Differences in Flavour Volatiles from Hybrid and Conventional Foxtail Millet Varieties Based on Gas Chromatography–Ion Migration Spectrometry and Chemometrics
Source: Plants (Basel). 2025 Feb 26;14(5):708. doi: 10.3390/plants14050708 (PMC11902185; doi:10.3390/plants14050708)
Supplement: Supplementary file 1 [file plants-14-00708-s001.zip › Supplementary Figure S1.pdf]

Supplementary Figure S1

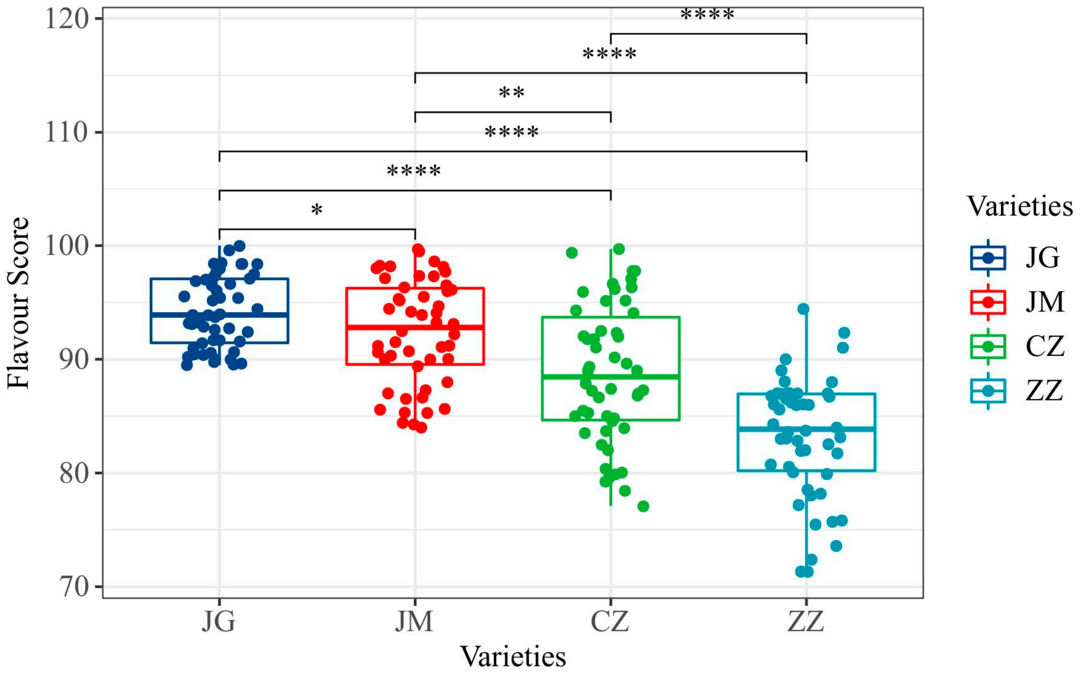

Supplementary Figure S1. Flavour scores of different varieties of foxtail millet
