# Supplementary material for: Uncovering the Differences in Flavour Volatiles from Hybrid and Conventional Foxtail Millet Varieties Based on Gas Chromatography–Ion Migration Spectrometry and Chemometrics
Source: Plants (Basel). 2025 Feb 26;14(5):708. doi: 10.3390/plants14050708 (PMC11902185; doi:10.3390/plants14050708)
Supplement: Supplementary file 1 [file plants-14-00708-s001.zip › Table S1.pdf]

Supplemental Table S1 Volatile Organic Compounds Classification and Related Data

NO	Chemicals	Classification	Retention index	Retention time/s	Drift time/ ms	Relative amount/%			
						JG	JM	ZZ	CZ
1	benzaldehyde	Aldehyde	1504	976.529	1.15543	0.50±0.02 b	0.49±0.02 b	0.60±0.03 a	0.42±0.02 c
2	Acetic acid	Organic Acid	1476.1	915.682	1.05313	1.81±0.01 b	1.18±0.03 c	0.82±0.00 d	2.29±0.02 a
3	1-Octen-3-ol-D	Alcohol	1464	890.354	1.16293	0.74±0.00 b	0.35±0.01 d	0.88±0.02 a	0.57±0.01 c
4	1-Octen-3-ol-M	Alcohol	1464	890.354	1.60547	4.33±0.07 a	3.51±0.01 b	4.30±0.11 a	4.38±0.05 a
5	(E)-2-octenal	Aldehyde	1429.9	822.736	1.33719	0.33±0.01 c	0.51±0.01 b	0.59±0.02 a	0.35±0.00 c
6	1-nonanal-D	Aldehyde	1400.8	769.199	1.47517	3.04±0.03 a	2.25±0.02 b	2.13±0.01 c	1.89±0.02 d
7	1-nonanal-M	Aldehyde	1401.1	769.844	1.94523	4.86±0.04 a	4.24±0.05 c	3.80±0.02 d	4.43±0.04 b
8	1-hexanol-D	Alcohol	1375.4	725.311	1.32949	0.26±0.00 c	0.37±0.01 b	0.79±0.01 a	0.27±0.01 c
9	1-hexanol-M	Alcohol	1375.4	725.311	1.64222	1.36±0.01 c	1.49±0.01 b	2.20±0.01 a	1.49±0.01 b
10	6-methyl-5-hepten-2-one	Ketone	1356.9	694.954	1.17902	0.53±0.02 b	0.63±0.02 a	0.61±0.03 a	0.51±0.01 b
11	(E)-2-Heptenal-D	Aldehyde	1341.6	670.82	1.25746	2.06±0.02 c	3.07±0.03 a	2.86±0.02 b	1.44±0.01 d
12	(E)-2-Heptenal-M	Aldehyde	1341.8	671.11	1.67101	3.32±0.03 b	3.54±0.02 a	3.15±0.05 c	3.04±0.04 d
13	(2.6)-dimethylpyrazine	Nitrogen-containing Heterocyclic Compound	1328.1	650.162	1.14275	0.26±0.01 a	0.18±0.01 b	0.13±0.01 d	0.15±0.01 c
14	1-octanal-D	Aldehyde	1308.2	620.88	1.40205	1.81±0.02 a	1.46±0.01 b	1.03±0.03 d	1.21±0.02 c
15	1-octanal-M	Aldehyde	1308.2	620.88	1.82503	3.16±0.03 a	2.72±0.03 b	2.08±0.03 c	2.71±0.03 b
16	1-Pentanol-D	Alcohol	1268.1	550.058	1.25548	2.21±0.02 b	2.44±0.01 a	2.13±0.01 c	2.07±0.01 d
17	1-Pentanol-M	Alcohol	1267.3	548.61	1.51143	3.45±0.01 b	3.40±0.02 c	2.88±0.03 d	3.49±0.03 a
18	ethyl 2-oxopropanoate	Ester	1258	531.71	1.15215	0.83±0.02 c	1.1±0.02 a	1.01±0.02 b	0.54±0.03 d
19	2-pentyl furan	Furan Compound	1242.9	505.377	1.2561	6.00±0.08 c	6.95±0.12 b	8.56±0.09 a	7.07±0.11 b
20	(E)-2-hexen-1-al-D	Aldehyde	1234.3	490.965	1.18403	0.93±0.01 c	1.38±0.00 a	1.34±0.02 b	0.68±0.01 d
21	(E)-2-hexen-1-al-M	Aldehyde	1233	488.964	1.52077	1.54±0.01 b	1.70±0.01 a	1.32±0.00 d	1.42±0.00 c

22	3-Methyl-1-butanol	Alcohol	1222.4	471.708	1.24493	0.59±0.01 d	1.00±0.01 c	1.31±0.01 a	1.09±0.01 b
23	Heptaldehyde-D	Aldehyde	1199.4	436.609	1.33139	5.40±0.04 a	3.76±0.04 b	2.43±0.02 d	3.34±0.02 c
24	Heptaldehyde-M	Aldehyde	1198.1	434.711	1.69727	3.31±0.06 a	2.61±0.03 c	1.99±0.02 d	2.94±0.01 b
25	1-Penten-3-ol	Alcohol	1174.9	400.919	0.94402	1.62±0.02 b	1.68±0.00 a	1.53±0.01 c	1.65±0.01 a
26	(E)-2-Pentenal-D	Aldehyde	1148.6	365.148	1.10827	0.48±0.00 c	0.74±0.01 a	0.55±0.01 b	0.44±0.01 d
27	(E)-2-Pentenal-M	Aldehyde	1148.2	364.655	1.36076	0.77±0.01 c	0.91±0.00 a	0.65±0.00 d	0.8±0.01 b
28	1-hexanal	Aldehyde	1101.9	309.422	1.565	6.70±0.34 b	7.49±0.22 a	6.77±0.36 b	7.77±0.43 a
29	(E)-2-Butenal-D	Aldehyde	1064	275.047	1.03763	0.11±0.00 c	0.16±0.01 b	0.18±0.00 a	0.09±0.00 d
30	(E)-2-Butenal-M	Aldehyde	1063.7	274.826	1.20406	0.41±0.02 a	0.46±0.03 a	0.44±0.05 a	0.35±0.02 b
31	1-Propanol	Alcohol	1058.1	270.185	1.11579	0.20±0.01 a	0.20±0.01 a	0.19±0.03 a	0.2±0.02 a
32	2-methyl-1-propyl acetate	Ester	1029.3	247.536	1.23429	0.09±0.01 a	0.07±0.01 b	0.07±0.00 b	0.08±0.01 b
33	2-butanol	Alcohol	1018.6	239.669	1.15379	0.10±0.01 ab	0.09±0.01 b	0.06±0.01 c	0.11±0.01 a
34	2-Pentanone	Ketone	997.1	224.498	1.36991	0.45±0.01 c	0.46±0.01 c	0.73±0.02 a	0.58±0.01 b
35	n-Pentanal	Aldehyde	998.2	225.2	1.42766	4.99±0.06 b	5.09±0.08 b	4.43±0.05 c	5.31±0.11 a
36	3-methyl-2-pentanone	Ketone	1009.3	232.926	1.47316	0.98±0.03 c	1.02±0.02 c	1.16±0.01 a	1.12±0.02 b
37	Ethanol-M	Alcohol	968.6	208.27	1.04506	3.57±0.05 c	3.66±0.05 b	3.28±0.03 d	4.24±0.04 a
38	Ethanol-D	Alcohol	951.2	199.043	1.12972	2.64±0.02 b	2.75±0.01 a	2.39±0.01 c	2.77±0.01 a
39	2-Butanone	Ketone	916.4	181.727	1.25013	2.41±0.09 a	2.02±0.05 b	2.54±0.06 a	2.48±0.06 a
40	Acetic acid ethyl ester	Ester	930	188.287	1.3298	1.34±0.02 b	1.26±0.02 d	1.30±0.01 c	1.44±0.02 a
41	3-Methyl butanal	Aldehyde	930	188.287	1.40745	0.47±0.01 c	0.62±0.02 b	0.81±0.01 a	0.84±0.02 a
42	Butanal	Aldehyde	891.3	170.201	1.28591	0.64±0.02 c	0.64±0.02 c	0.98±0.02 a	0.73±0.02 b
43	2-propanone	Ketone	834.2	146.618	1.11981	10.71±0.15 b	10.08±0.16 c	9.43±0.16 d	11.08±0.2 a
44	Propanal	Aldehyde	808.3	137.043	1.14141	1.36±0.05 b	1.35±0.07 b	1.42±0.04 b	1.65±0.11 a
45	(Z)-furan linalool oxide-D	Terpenes and Terpenoids	1414.2	793.365	1.26345	0.47±0.01 c	0.51±0.01 b	0.56±0.01 a	0.27±0.01 d
46	(Z)-furan linalool oxide-M	Terpenes and Terpenoids	1412.4	790.188	1.81287	0.86±0.02 c	1.26±0.01 b	1.35±0.00 a	0.80±0.01 d
47	2-Octanone-D	Ketone	1300.1	609.344	1.33623	0.19±0.00 d	0.31±0.01 b	0.86±0.01 a	0.29±0.01 c

48	2-Octanone-M	Ketone	1300.8	610.349	1.75856	0.65±0.01 d	0.76±0.01 c	1.14±0.02 a	0.84±0.01 b
49	Ethyl hexanoate	Ester	1250.2	518.067	1.33512	0.25±0.01 c	0.25±0.01 c	0.30±0.01 a	0.28±0.01 b
50	3-Methyl-2-butenal-D	Aldehyde	1215.7	461.283	1.09296	0.15±0.00 a	0.13±0.00 b	0.12±0.00 c	0.14±0.01 ab
51	3-Methyl-2-butenal-M	Aldehyde	1215.1	460.4	1.36187	0.35±0.01 b	0.33±0.01 c	0.22±0.01 d	0.38±0.01 a
52	2-Heptanone	Ketone	1193.3	427.742	1.63501	2.78±0.01 c	3.80±0.02 b	6.05±0.15 a	3.93±0.02 b
53	1-butanol-D	Alcohol	1159.9	380.051	1.18087	0.09±0.01 b	0.10±0.01 a	0.12±0.01 a	0.08±0.01 b
54	1-butanol-M	Alcohol	1158.9	378.762	1.37666	0.42±0.01 b	0.43±0.01 b	0.47±0.01 a	0.47±0.01 a
55	2-ethyl hexanol	Alcohol	1521.1	1015.946	1.41443	0.16±0.02 c	0.23±0.01 b	0.29±0.01 a	0.12±0.02 d
56	Decanal	Aldehyde	1494.2	954.836	1.53898	0.35±0.01 a	0.26±0.01 b	0.23±0.02 c	0.24±0.02 c
57	(E.E)-2.4-heptadienal	Aldehyde	1484.5	933.496	1.19206	0.11±0.01 b	0.15±0.01 a	0.10±0.01 bc	0.08±0.02 c
58	(E)-3-penten-2-one	Ketone	1114.5	323.619	1.09344	0.16±0.01 a	0.15±0.00 a	0.12±0.00 b	0.17±0.00 a
59	2-methyl-1-Propanol	Alcohol	1112.3	321.042	1.17119	0.32±0.00 b	0.27±0.00 c	0.25±0.00 d	0.38±0.00 a
